# Compliance Behaviour After a Coronary Ischaemic Event: A Quasi-Experimental Study of Adherence to a Protocolised Follow-Up in Primary Care

**DOI:** 10.3390/jcdd11120407

**Published:** 2024-12-19

**Authors:** Ángel Lizcano-Álvarez, Laura Carretero-Julián, Ana Talavera-Sáez, Almudena Alameda-Cuesta, Rocío Rodríguez-Vázquez, Beatriz Cristobal-Zárate, María-Gema Cid-Expósito

**Affiliations:** 1 Faculty of Health Sciences, Department of Nursing and Stomatology, Rey Juan Carlos University, Alcorcón, 28922 Madrid, Spain; angel.lizcano@urjc.es (Á.L.-Á.); ana.talavera@urjc.es (A.T.-S.); almudena.alameda@urjc.es (A.A.-C.); rocio.vazquez@urjc.es (R.R.-V.); 2 Nursing Research Group Cardiovascular Care, Madrid Society of Family and Community Nursing (SEMAP), 28007 Madrid, Spain; mcarre20@ucm.es (L.C.-J.); bea_zafiro@hotmail.com (B.C.-Z.); 3 Faculty of Nursing, Physiotherapy and Podiatry, Department of Nursing, Complutense University, 28040 Madrid, Spain; 4 Barcelona Healthcare Centre, Primary Care Assistance Management, Madrid Health Service, Móstoles, 28933 Madrid, Spain

**Keywords:** cardiovascular diseases, myocardial infarction, patient compliance, cardiovascular risk factors, primary health care, nursing, self care

## Abstract

Following a coronary ischaemic event, it is essential to promote empowerment in self-care decision making. Primary care nursing is crucial for intensive follow-up to promote adherence to the therapeutic regimen. Objective: To ascertain whether adherence to a protocolised follow-up programme, with the support of a patient notebook, improves compliance behaviours in terms of physical activity, prescribed diet and medication. This is a quasi-experimental multicentre pre/post study. Population: Individuals aged 40–70 years, diagnosed with cardiac ischaemia in the last 18 months with a follow-up from March 2017 to January 2019, were included in a protocolised follow-up programme consisting of 11 visits over 12 months. A total of 194 patients started the programme and 132 completed it. Of these, 67.4% exhibited good adherence to follow-up, 31.8% exhibited medium adherence, and 0.8% exhibited poor adherence. Therefore, the patients were recoded into two variables: Medium–Low Adherence and High. The Nursing Outcomes Classification variables were significantly different between the Poor–Medium and Good Adherence groups and were always higher in the Good Adherence group (*p*-values < 0.05 t-student). There was a significant relationship between level of adherence and compliance behaviour. Good adherence to a follow-up plan led by primary care nurses improves compliance behaviours in terms of prescribed diet, physical activity, and medication. Early, intensive and protocolised follow-up by primary care nurses is essential to improve adherence to the therapeutic regimen and compliance behaviour among individuals with cardiac ischaemia. The use of a cardiovascular self-care notebook promotes adherence.

## 1. Introduction

The onset of cardiovascular disease (CVD) can be prevented, and the consequences of the disease may be minimized by the management of cardiovascular risk factors, many of which are behavioural in nature [1]. However, the EUROASPIRE V study, conducted in 27 European countries, shows that the vast majority of individuals with CVD lead unhealthy lives in terms of smoking, diet, sedentary lifestyle, and poor adherence to their treatment plans [2]. Early initiation and long-term maintenance of secondary prevention programmes are required to improve adherence to the treatment plan and to achieve lifestyle changes [3].

Adherence to the treatment plan involves a set of individual actions and behaviours in line with professional recommendations, such as adherence to medication, dietary regimen, or a follow-up plan. However, individuals with CVD have difficulty in maintaining adherence, which decreases considerably six months after discharge from hospital [3,4,5,6].

Amongst patients, the underlying causes can be attributed to low self-efficacy, a lack of motivation to learn about the prevention and control of CVD, a limited understanding of the subject matter, and beliefs about the health-disease process [4,7]. Therefore, continuous assessment of patient compliance behaviour is required. The Nursing Outcomes Classification (NOC) [8] is a standardised terminology for practice-sensitive outcomes in nursing. NOC outcomes include Compliance Behaviour: Prescribed Diet (1622), Compliance Behaviour: Prescribed Medication (1623) and Compliance Behaviour: Prescribed Activity (1632), which are fundamental axes in the follow-up of individuals with cardiac ischaemia. The NOC defines these outcomes as voluntary personal actions to follow a therapeutic regimen prescribed by health professionals.

Consequently, the individual should abandon the typical passive role and adopt an active role, with a good level of self-care and self-management of their health-disease process, participating in decision making and committing themselves to their own health [5,9,10]. Hence the importance of the role of accountability in adherence programmes. There is a link between adherence and accountability, as intensive follow-up during the first months after an ischaemic event improves the therapeutic relationship given that patients are accountable to their healthcare professional [11].

Primary care nurses are responsible and referents for the secondary cardiovascular prevention of patients with cardiac ischemia after hospital discharge. The primary care model in Spain has a structure based on health centres, each with a multidisciplinary team made up of general practitioners, paediatricians and nurses. Through health education and continuous follow-up, primary care nurses support the empowerment of patients regarding their cardiovascular self-care [9,12,13].

To improve shared decision making, primary care nursing care is based on the use of taxonomies such as NANDA-International [14], NOC [8], and NIC (Nursing Interventions Classification) [15]. In addition, an individual and family approach is adopted, favouring an integrated and holistic approach to the patient. Furthermore, the assessment of M. Gordon’s functional patterns [16] helps nurses to systematise the nursing care they provide to patients with CVD [17,18,19].

A number of studies have demonstrated the superior efficacy of nurse-led cardiovascular prevention programmes, both for patients with established CVD and for high-risk individuals, in comparison to the conventional approach [20]. However, it is important that secondary cardiovascular prevention is implemented as soon as possible after hospital discharge, as it improves quality of life and reduces readmissions and mortality [21].

To foster compliance behaviours among CVD patients, it is necessary to support them with tools to motivate them and facilitate follow-up in primary care nursing consultations. The Madrid Society of Family and Community Nursing (SEMAP), along with six Spanish nursing societies, developed the Patient’s Notebook in heart-healthy self-care [22]. This notebook is intended to improve communication between nurses and patients, to foster patient and family involvement in the self-management of risk factors through self-reporting, to increase patient knowledge, and to facilitate the follow-up plan and communication between professionals in healthcare facilities and those in the hospital setting. It also promotes patient empowerment and follow-up by primary care professionals [23].

Consequently, our objective was to ascertain whether good adherence of patients with cardiac ischaemia to a protocolised therapeutic follow-up, led by primary care nurses and with the support of the cardiovascular self-care notebook, could improve the following NOC outcomes: Compliance Behaviour: Prescribed Diet (1622), Compliance Behaviour: Prescribed Activity (1632), and Compliance Behaviour: Prescribed Medication (1623).

## 2. Materials and Methods

### 2.1. Design

This is a quasi-experimental multicentre pre/post study without a control group. The results of the study are reported in compliance with the Transparent Reporting of Evaluations with Non-randomised Designs (TREND) statement [24].

### 2.2. Setting and Participants

The study was conducted from March 2017 to January 2019 in nurses’ offices of 40 healthcare facilities in Madrid (Spain). The inclusion criteria were patients between 40 and 70 years old who were diagnosed in the previous six months with one of the following ICPC diagnoses: K74 (Ischaemic heart disease with angina); K75 (Acute myocardial infarction); or K76 (Ischaemic heart disease without angina) [25]. Along with the informed consent, patients signed an agreement to attend all appointments for the duration of the programme. Patients with a life expectancy below one year, with problems travelling independently, and with comprehension difficulties due to cognitive impairment or language barriers were excluded from the programme.

### 2.3. Recruitment

An informative e-mail was sent to 320 primary care nurses inviting them to participate in the study. Of these, 59 nurses showed interest in participating in the study by signing a research commitment document. This group of nurses was referred to as the Clinical Care Group.

A non-probability purposive sampling technique was used by selecting patients who attended consultations at their healthcare facilities and met the inclusion criteria. Each nurse was asked to select at least three patients for follow-up. The recruitment period lasted five months (from February to June 2017).

**Figure 1:**
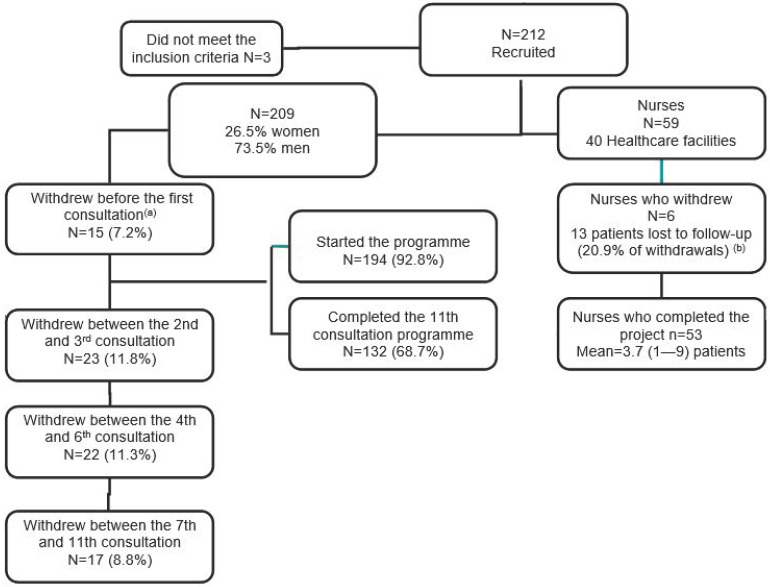
Flow chart showing the study population and recruited nurses. ^a^ Patients who dropped out of the study after signing the informed consent form at consultation 0 and did not attend the first consultation; ^b^ percentage of the 62 patients who dropped out once the programme had started.

The sample size required to estimate an unknown prevalence from a finite population size with 50% variance, 95% confidence and 5% precision was set at 341 patients. Due to time constraints during the recruitment period, the study was ultimately initiated with a sample of 209 patients (Figure 1).

### 2.4. Variables

Data on sociodemographic and clinical variables were recorded. The sub-variables relating to the level of adherence to the protocol were based on the register made by the nurses at the beginning of each consultation and were as follows: (1) whether or not the patient attended the new appointment in accordance with the protocol; (2) whether or not the patient self-recorded data and used the self-care notebook; and (3) whether or not the patient complied with the recommendations provided to them at the previous visit. Each was awarded between 1 and 3 points, from lowest to highest compliance or adherence. The three sub-variables were recoded into one main variable: overall adherence to the monitoring plan (OAMP). To calculate the OAMP, the three sub-variables were measured during ten of the eleven visits of the follow-up protocol (2nd to 11th), making the OAMP range 30–90 points.

Therefore, the outcome variables were the OAMP and the following NOC outcome indicators: Compliance Behaviour: Prescribed Medication (1623), Compliance Behaviour: Prescribed Activity (1632) and Compliance Behaviour: Prescribed Diet (1622) (Table 1). Each indicator was rated on a Likert scale ranging from 1 (never demonstrated) to 5 (always demonstrated).

### 2.5. Ethical Considerations

The current study was conducted after receiving ethical approval from the Institutional Review Board of the Hospital XXX Clinical Research Ethics Committee (approval nº 13X/1X) and followed the ethical principles contained in the Declaration of Helsinki [26].

Each nurse was responsible for ensuring that participating patients were adequately informed about the nature, purpose, risks and benefits of the study. Prior to the commencement of data collection, informed consent was obtained and signed by both nurses and patients.

The data were entered into a database and used exclusively by the research team in accordance with Spanish legislation (Organic Law 15/1999 of 13 December for Protection of Personal Data; and Law 14/2007 of 3 July for Biomedical Research).

### 2.6. Intervention and Data Collection Procedures

To optimize the standardization of the intervention and the use of the cardiovascular self-care notebook, nurses were taught to standardize the recording of data and intervention procedures through a manual that protocolised the actions to be carried out in each nursing consultation. This was recorded in the patient’s medical record.

The organizational structure (Figure S1) was nodal and consisted of three levels. The first level, the Technical Research Group (TRG), was made up of two main researchers and two coordinators. These coordinators were responsible for monitoring four nodes of nurses. In each of these four nodes, there were nurses who would form the second level, called the Clinical Research Group (CRG). Each of these eight nurses was responsible for the follow-up of one node, the third level, formed between seven and eight nurses, who, as mentioned above, constituted the Clinical Care Group (CCG).

Patients who were initially interested were scheduled for the recruitment appointment or informative consultation. The study was explained to them and those interested signed the informed consent form and were then given the cardiovascular self-care notebook.

Eleven interventions were performed in nursing consultations over a period of 12–15 months. For all patients, each consultation was assigned a frequency, the first ones being more frequent (15 days) to favour the therapeutic relationship, while the last ones were at an interval of between 30 and 45 days. The estimated duration of each consultation was between 30 and 60 min.

There were actions common to each intervention including the three sub-variables of the overall adherence to the monitoring plan (OAMP):

- Assessment of adherence to the follow-up plan and self-care plan from the previous consultation (whether or not the patient attended the new appointment in accordance with the protocol and complied with the recommendations provided to them at the previous visit).
- Actions with the patient. Assessment and data collection on the NOC indicators linked to the corresponding M. Gordon’s functional health pattern.
- Actions with the patient’s notebook, including a self-monitoring section and a cardiovascular risk factor (CVRF) self-care information section (whether or not the patient self-recorded data and used the self-care notebook).
- Recording of information on the data collection platform and the medical record.
- Explaining heart-healthy actions and self-care to the patient for the next consultation.

The structure of the follow-up protocol in each nursing consultation followed M. Gordon’s functional health patterns. This was as follows:

- Consultation 0: Recruitment consultation. Signing the informed consent form.
- Consultation 1: History. Heart-healthy lifestyle.
- Consultation 2: Assessment of the health perception/health management pattern and the cognitive-perceptual pattern.
- Consultation 3: Assessment of the nutritional-metabolic pattern.
- Consultation 4: Assessment of the nutritional-metabolic pattern.
- Consultation 5: Assessment of the physical activity/exercise pattern.
- Consultation 6: Assessment of the self-perception/self-concept pattern.
- Consultation 7: Assessment of the role-relationship pattern and sexuality-reproductive pattern.
- Consultation 8: Assessment of the coping/stress tolerance pattern and value-belief pattern.
- Consultation 9: Assessment of the elimination pattern and sleep-rest pattern.
- Consultation 10: Family assessment.
- Consultation 11: Final assessment. Expert Patient Diploma.

During each consultation, both the OAMP and the assessments of the NOC indicators associated with each of M. Gordon’s functional patterns were documented [16]. In the final consultation, a final assessment of all the NOC indicators was made. To minimise the bias of feeling observed and evaluated, which could influence the final assessment, the nurse could not see the assessments or scores assigned to the indicators in previous consultations.

### 2.7. Data Analysis

To analyse the potential pre-post variation in the NOC dimensions and whether they varied similarly depending on the level of adherence, a general linear model of repeated measures was applied.

To verify the reliability of the NOC indicators, Cronbach’s α internal consistency model [27] was used, considering values ≥0.7 as optimal. To confirm the one-dimensionality of the NOC indicators and the validity of the scales, an exploratory factor analysis of principal components with Varimax rotation was applied using the criterion of eigenvalues greater than 1. Significance was ensured using the Kaiser–Meyer–Olkin test and Bartlett’s test of sphericity.

The Student’s *t*-test for two independent samples (parametric) and the one-way ANOVA for more than two independent samples (parametric) were also used. Pearson’s correlation was used for continuous variables. Given the adequate sample size in each group (n > 30), normality of the main variables was assumed by applying the central limit theorem [28]. The statistical significance threshold was set at 5% (α = 0.05). The data were processed and analysed using SPSS (version 25) software by IBM.3. Results

Of the 209 patients who signed the consent form, 194 started the programme (92.8%) and 132 (68.7%) completed the 11 consultations. The follow-up between the first consultation (PRE) and the last (POST) was on average 356.10 days (SD 25.4). The interval between each consultation was 32.37 days (SD 2.3).

Of these 132 patients, 73.5% were male, with a mean age of 58.2 (SD = 9.7) years; the mean age of the women was 61.4 years (SD = 16.2). The percentage of men between 40 and 55 years old was 35.1%, while the percentage of women in this premenopausal and menopause range was lower at 28.6%. In addition, 52.3% had completed secondary or tertiary education. Their cardiovascular risk factors included the following: hypertension (58.3%); dyslipidaemia (65.2%); diabetes (24.2%); obesity (32.1%); and tobacco use (10.6%).

The mean adherence for the OAMP variable was 80.8% (SD = 10.9). This level of adherence was divided into quartiles, resulting in three levels: less than 50 points (poor adherence), between 50 and 75 points (moderate adherence), and between 76 and 100 points (good adherence). Thus, 67.4% had good adherence, 31.8% had moderate adherence and only 0.8% had poor adherence. This led to a recording of the levels into two variables: Moderate–Low Adherence and High Adherence.

Regarding the relationship between sociodemographic and clinical variables with the OAMP (Table 2), the only variable with statistical significance was tobacco use (** *p* < 0.001). Individuals who smoked at the time of the study had a lower OAMP than ex-smokers and non-smokers. On analysis of the relationship between sociodemographic and clinical variables with adherence by levels (Moderate–Low Adherence and High Adherence) (Table 3), high adherence seems to be linked to patients who do not currently smoke (** *p* < 0.001). There was a tendency for men to have better adherence than women (* *p* < 0.052). The remaining variables did not have a significant relationship with the various adherence levels.

With the exception of the post value of Compliance Behaviour: Prescribed Medication (close to the threshold of 0.7), the criteria for reliability as a measurement scale can be considered to be met. An exploratory factor analysis was performed on all results to assess their one-dimensionality, both pre and post. All results were valid (KMO > 0.6 and Bartlett’s sphericity *p* < 0.05) and one-dimensional (one single factor with eigenvalue > 1), while the percentage of variance explained was greater than 40% [29,30]. To assess the relationship between the OAMP and the three compliance behaviour outcomes (medication, diet, and physical activity), we analysed whether there were variations between pre and post, and whether these variations occurred in line with the level of adherence (Table 4).

Our results reflect a significant change in the adherence indicators throughout follow-up (pre-post). The two levels of adherence were the same for adherence to diet (mean +0.45) and for adherence to physical activity (mean +0.33). By contrast, compliance with medication showed a greater increase in the group with lower adherence (0.88 points) than in the group with high adherence (0.64 points).

## 3. Discussion

Adherence to treatment regimens is one of the most influential factors in chronic diseases. Indicators of adherence include patient attendance to scheduled appointments with healthcare professionals, compliance with the recommendations made by these professionals, and their assessment of the implementation of these recommendations. Given that accountability favours responsible behaviour in adherence programmes [31], and that most adherence interventions occur during visits to professionals, we found that the use and evaluation of the notebook as a health education tool at the beginning of each of the 11 consultations favoured follow-up and promoted shared decision making. The use of patient self-management guidelines and improved cardiovascular care skills among PHC nurses are also influential factors in improving adherence [32].

Adherence may have a multifactorial influence. Firstly, it is important to consider the role of sex/gender as predictors of higher adherence in men and lower adherence in women in secondary prevention. These determinants should be taken into account in our clinical practice [32,33,34].

The observed correlation between male sex and enhanced adherence is corroborated by the findings of the studies conducted by Huber et al. [35] and Moreno et al. [36], where female sex was identified as a predictor of non-adherence to medication, diet and physical activity. It is notable that female sex itself was not identified as a biological predictor of non-adherence; rather, it was aspects related to gender inequalities, such as family care burden, depression or smoking, that emerged as significant factors [34].

Educating cardiovascular patients through structured education with telephone follow-up and monitoring has a statistically significant positive effect on self-care, self-efficacy and quality of life and thus adherence to the treatment plan [37]. Our data on improved self-care in association with intensive nurse-led follow-up among patients with ischaemic heart disease are consistent with other studies [38]. In the RESPONSE-2 trial, the effect of comprehensive primary care nursing programmes on lifestyle was assessed [39]. Similarly, in a scoping review, Freeley et al. [32] confirm that there is a sustained improvement over time when health professionals provide care in a person-centred manner.

The higher overall adherence to intensive follow-up of our patients in both diet and physical activity, and consequently their improvement, correlates with the results of a meta-analysis concluding that educational interventions for secondary prevention are effective in improving healthy behaviours at short- and long-term follow-up, with longer duration programmes being more effective [40].

It is quite possible that a long-term relationship between nurse and patient, an involvement of nursing with continuous follow-up, improves adherence to diet, physical activity [41], and medication as a result of mutual trust and a better understanding of the disease [4,35].

Regarding diet and physical activity, as reported in the clinical trials by Zakeri et al. [4] and Köhler et al. [9], there is a clear emphasis on the significance of nurse-led educational programs in empowering patients to navigate their disease process and to adhere to dietary and physical activity regimens. In our study, as in the trial by Westland et al. [42], the support of primary care nurses promotes the effectiveness of engaging patients in heart-healthy physical activity and avoiding sedentary lifestyles [2].

One striking result of our study was a major change in compliance behaviour with prescribed medication in the Moderate–Low adherence group when compared with adherence to diet and physical activity. This could be due to a social preconception and certain coping behaviours of the individual in the face of a new infarction, as they perceive medication to be more important than following lifestyle recommendations or attending nurse’s appointments. Other studies link the benefits of joint decision making in an established patient–professional relationship to improved medication adherence [43].

Poor adherence to prescribed medication is one of the most recurrent themes in morbidity and mortality studies in cardiovascular prevention, as reflected in the results of the Euroaspire V study [2]. A strategy that would supplement our follow-up intervention in the future would be reinforcing adherence to the therapeutic plan by means of telephone or internet follow-up [44]. Although studies on the presence of accountability in digital interventions are still lacking [45], research has shown improvement in terms of adherence to prescribed diet and medication among patients with myocardial infarction [35]. In the study by Shim and Hwang, conducted with older people with acute myocardial infarction, it was found that there was an increase in patients’ quality of life after one year of telephone follow-up [12].

Improvements in this type of intervention may be required, which may include training primary care nurses in the development of skills for follow-up after a coronary event [23] or developing innovative programmes by interdisciplinary teams that address all aspects of lifestyle and CVRF management [2].

Our study supports the statement of the Association of Cardiovascular Nursing and Allied Professions of the European Society of Cardiology, which reaffirms the need for integrated and holistic cardiovascular care involving patients and families to ensure that the person is at the centre of care [45].

Limitations: the exclusion criteria used in this study were based on age and having a life expectancy of less than one year. As a result, our sample may be biased in favour of healthier patients. As this was a quasi-experimental study, there was no control group or randomisation. Although there was a training seminar for nurses and a data collection manual was made available, inter- and intra-observer variability may have been present. The OAMP variable and its levels were designed based on the experiential criteria of the researchers. In this study, as recommended by the NOC taxonomy itself, the indicators for each outcome were selected according to the care setting and the characteristics of the individuals. Nevertheless, this selection could have been validated on the basis of relevance to the primary care setting, the estimated duration of patient care, conceptual clarity, and understandability. Similarly, there may have been a drop-out bias due to the abandonment of the follow-up programme, which could be taken into account by other authors wishing to carry out similar research with such intensive and prolonged follow-up.

Therefore, our study shows that primary care nurses can undertake an intensive, protocolised and comprehensive follow-up of patients during the first months after a coronary ischemic event. The role of the primary care nurse enhances adherence to the therapeutic regimen, self-management of heart disease, and compliance with the prescribed diet, physical activity, and medication. The use of self-care-based health education tools improves the empowerment and self-efficacy of people with chronic cardiovascular disease and their family members.

Whether a limitation or a strength, we were unable to find any studies on the effects of an educational tool such as our patient notebook on improving follow-up, compliance and empowerment in patients with cardiac ischaemia. Finally, the follow-up period was one year, which means that we do not know whether the results obtained were maintained afterwards.

## 4. Conclusions

The results of this study suggest that a good level of adherence to a follow-up plan led by primary care nurses leads to improved compliance with self-care in terms of prescribed diet, physical activity, and medication. Furthermore, our results highlight the importance of secondary cardiovascular prevention programmes in primary care focusing on the interaction between patients and nurses, as this is considered to be one of the most important factors in improving the health of patients with chronic diseases such as CVD. The educational support of a cardiovascular self-care notebook leads to improved adherence to the therapeutic regimen.

## Figures and Tables

**Table 1:** Outcome indicators selected for each Nursing Outcomes Classification (NOC) outcome.

NOC Outcome	Outcome Indicators
1632 Compliance Behaviour: Prescribed Activity	Identifies expected benefits of physical activity.
	Identifies barriers to implement prescribed physical activity.
	Sets achievable short-term activity goals with health professional.
	Participates in prescribed physical activity (3–5 days per week, 30–45 min per day, or 150 min per week with intensity specific to each patient).
	Seeks external reinforcement for performance of health behaviours.
	Identifies and reports symptoms experienced during activity to health professional.
	Knows and monitors target heart rate set by health professional.
1622 Compliance Behaviour: Prescribed Diet	Plans and prepares heart-healthy meals consistent with activity and tastes.
	Knows what food to eat when eating out.
	Uses nutritional information on labels to guide selections.
	Close relatives are aware of the agreed diet.
	Participates in setting achievable dietary goals with health professional.
1623 Compliance Behaviour: Prescribed Medication	Keeps a list of all medication with dose and frequency.
	Obtains required medication.
	Takes all medication at intervals prescribed (assessed using the Morinsky–Green test).
	Knows and monitors medication therapeutic effects (why and for what purpose it is being taken).
	Knows and monitors medication side effects.
	Knows and informs health professional of all medication being taken (name, dosage, frequency, and how it is being taken).

**Table 2:** Relationship of variables to Overall Adherence Level (OAMP).

Variables		Overall Adherence Level (OAMP)		
		N (%)	M ± SD	(*p*-Value) *
Gender	Total	132 (100)	80.86 ± 10.99	0.167 ^1^
	Men	97 (73.5)	81.66 ± 10.92	
	Women	35 (26.5)	78.66 ± 11.05	
Level of education	Total	132 (100)	80.86 ± 10.99	0.969 ^2^
	Primary education	63 (47.7)	80.78 ± 11.68	
	Secondary education	49 (37.1)	81.14 ± 10.22	
	University education	20 (15.2)	80.45 ± 11.11	
ICPC	Total	132 (100)	80.86 ± 10.99	0.904 ^2^
	K74	47 (35.6)	80.70 ± 11.68	
	K75	82 (62.1)	80.85 ± 10.78	
	K76	3 (2.3)	83.67 ± 7.57	
High blood pressure	Total	132 (100)	80.86 ± 10.99	0.392 ^1^
	No	55 (41.7)	81.84 ± 10.53	
	Yes	77 (58.3)	80.17 ± 11.33	
Tobacco use	Total	132 (100)	80.86 ± 10.99	0.001 ***^2^
	Smoker	14 (10.6)	71.14 ± 10.47	
	Ex-smoker	58 (43.9)	80.53 ± 10.87	
	Non-smoker	60 (45.5)	83.45 ± 10.03	
Dyslipidaemia	Total	132(100)	80.86 ± 10.99	0.652 ^1^
	No	46 (34.8)	81.46 ± 10.76	
	Yes	86 (65.2)	80.55 ± 11.16	
Diabetes	Total	132 (100)	80.86 ± 10.99	0.624 ^1^
	No	100 (75.8)	81.13 ± 10.84	
	Yes	32 (24.2)	80.03 ± 11.60	
Body Mass Index	Total	132 (100)	80.86 ± 10.99	0.197 ^2^
	Normal (18.5–24.9)	23 (17.4)	77.83 ± 14.21	
	Overweight (25–29.9)	68 (51.5)	82.26 ± 10.60	
	Obese I (30–34.9)	27 (20.5)	81.85 ± 8.61	
	Obese II (35–39.9)	14 (10.6)	77.14 ± 10.22	

SD: standard deviation; ICPC: International Classification of Primary Care; K74: Ischaemic heart disease with angina; K75: Acute myocardial infarction; K76: Ischaemic heart disease without angina. ^1^ (Student’s *t*); ^2^ (one-way ANOVA) *** *p* < 0.001, two-tailed.

**Table 3:** Relationship of variables to Overall Adherence (OAMP) by levels.

Variables	Overall Adhesion (OAMP) by Levels				
		Total	Moderate–Low Adherence	High Adherence	(*p*-Value) *
		N	n (%)	n (%)	
Gender	Total	132	43 (32.6)	89 (67.4)	
	Men	97	27 (27.8)	70 (72.2)	0.052 ^1^
	Women	35	16 (45.7)	19 (54.3)	
Level of education	Total	132	43 (32.6)	89 (67.4)	
	Primary education	63	26 (41.3)	37 (58.7)	0.110 ^2^
	Secondary education	49	13 (26.5)	36 (73.5)	
	University education	20	4 (20.0)	16 (80.0)	
High blood pressure	Total	132	43 (32.6)	89 (67.4)	
	No	55	16 (29.1)	39 (70.9)	0.472 ^1^
	Yes	77	27 (35.1)	50 (64.9)	
Tobacco use	Total	132	43 (32.6)	89 (67.4)	
	Smoker	14	10 (71.4)	4 (28.6)	0.001 ***^2^
	Ex-smoker	58	20 (34.5)	38 (65.5)	
	Non-smoker	60	13 (21.7)	47 (78.3)	
ICPC	Total	132	43 (32.6)	89 (67.4)	
	K74	47	17 (36.2)	30 (63.8)	0.803 ^2^
	K75	82	25 (30.5)	57 (69.5)	
	K76	3	1 (33.3)	2 (66.7)	
Alcohol consumption	Total	132	43 (32.6)	89 (67.4)	
	No	7	3 (42.9)	4 (57.1)	0.551 ^1^
	Yes	125	40 (32.0)	85 (68.0)	
Dyslipidaemia	Total	132	43 (32.6)	89 (67.4)	
	No	46	14 (30.4)	32 (69.6)	0.701 ^1^
	Yes	86	29 (33.7)	57 (66.3)	
Diabetes	Total	132	43 (32.6)	89 (67.4)	
	No	100	30 (30.0)	70 (70.0)	0.264 ^1^
	Yes	32	13 (40.6)	19 (59.4)	
Body Mass Index	Total	132	43 (32.6)	89 (67.4)	
	Normal (18.5–24.9)	23	10 (43.5)	13 (56.5)	0.223 ^2^
	Overweight (25–29.9)	68	21 (30.9)	47 (69.1)	
	Obese I (30–34.9)	27	5 (18.5)	22 (81.5)	
	Obese II (35–39.9)	14	7 (50.0)	7 (50.0)	

ICPC: International Classification of Primary Care; K74: Ischaemic heart disease with angina; K75: Acute myocardial infarction; K76: Ischaemic heart disease without angina. ^1^ (Student’s *t*); ^2^ (one-way ANOVA) *** *p* < 0.001, two-tailed.

**Table 4:** Variation in adherence behaviour per adherence levels at the beginning (Pre) and end (Post) of the monitoring plan.

Compliance Behaviour	Level of Adherence	Pre	Post	Pre-Post Difference	
		M ± SD	M ± SD	M ± SD	*p*-Value *
Prescribed Medication	Total	3.98 ± 0.59	4.70 ± 0.37	0.71 ± 0.62	0.000 ***
	Moderate–Low Adherence	3.70 ± 0.66	4.58 ± 0.44	0.87 ± 0.68	0.039
	High Adherence	4.11 ± 0.50	4.75 ± 0.31	0.63 ± 0.58	
Prescribed Diet	Total	3.70 ± 0.68	4.14 ± 0.76	0.44 ± 0.83	0.000 ***
	Moderate–Low Adherence	3.30 ± 0.70	3.76 ± 0.80	0.46 ± 0.86	0.863
	High Adherence	3.89 ± 0.57	4.33 ± 0.67	0.43 ± 0.82	
Prescribed Activity	Total	3.99 ± 0.75	4.32 ± 0.67	0.33 ± 0.73	0.000 ***
	Moderate–Low Adherence	3.56 ± 0.78	3.89 ± 0.70	0.33 ± 0.82	0.962
	High Adherence	4.20 ± 0.64	4.53 ± 0.55	0.33 ± 0.69	

SD: standard deviation; Pre-post difference: at the beginning (Pre) and end (Post) of the monitoring plan. *** *p* < 0.001, two-tailed.

## Data Availability

The data presented in this study are available on request from the corresponding author. The data are not publicly available due to legal restrictions.

## Supplementary Materials

The following supporting information can be downloaded at: https://www.mdpi.com/article/10.3390/jcdd11120407/s1. Figure S1: Clinical Investigators group ReccAP.
