# Supplementary material for: Compliance Behaviour After a Coronary Ischaemic Event: A Quasi-Experimental Study of Adherence to a Protocolised Follow-Up in Primary Care
Source: J Cardiovasc Dev Dis. 2024 Dec 19;11(12):407. doi: 10.3390/jcdd11120407 (PMC11677688; doi:10.3390/jcdd11120407)

Clinical Investigators group REccAP

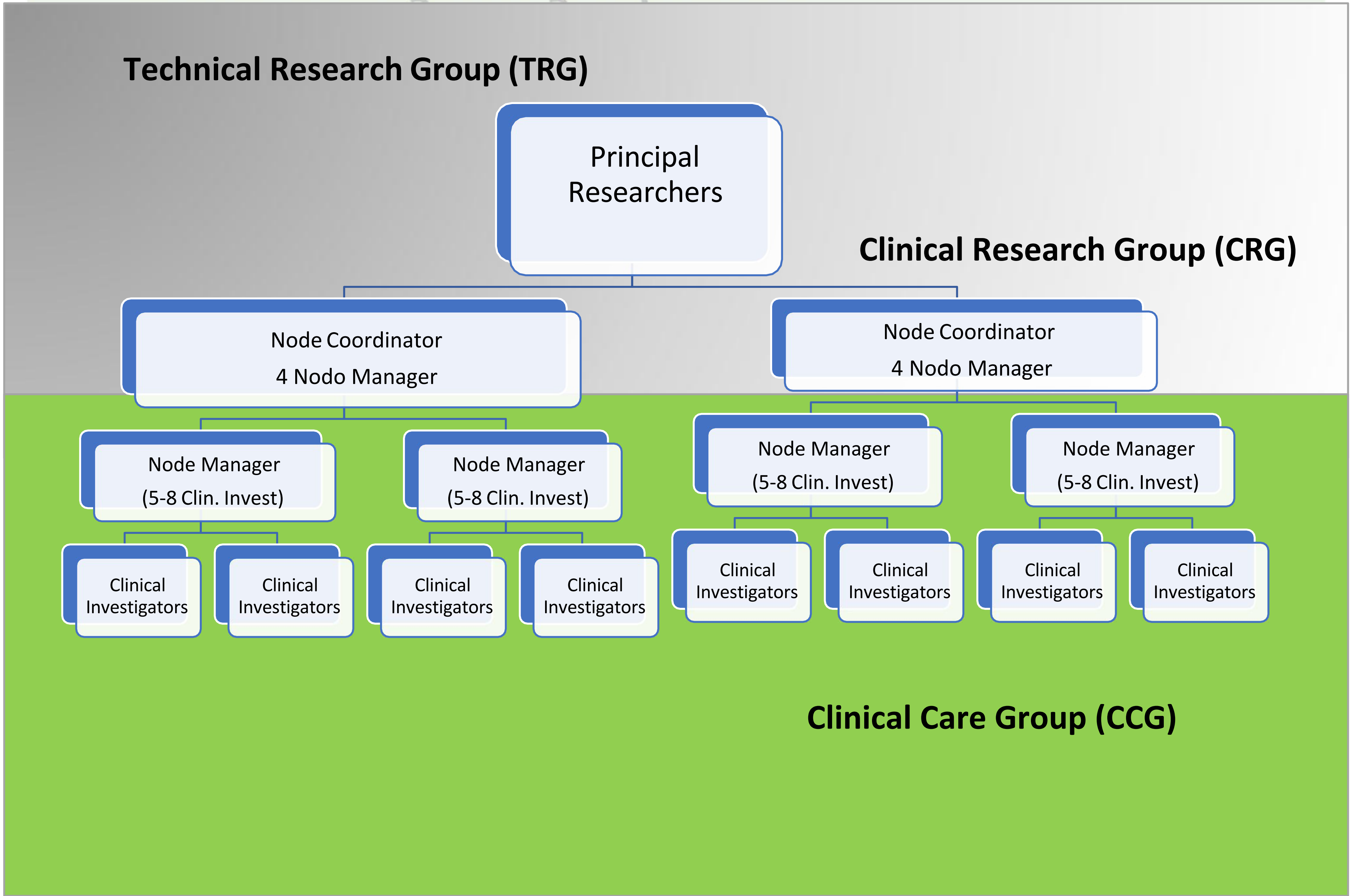

Supplement: Supplementary file 1 [file jcdd-11-00407-s001.zip › jcdd-3257128-supplementary.pdf]
